# Content analysis of digital media coverage of the human papillomavirus vaccine school-entry requirement policy in Puerto Rico

**DOI:** 10.1186/s12889-021-11311-9

**Published:** 2021-07-01

**Authors:** Vivian Colón-López, Vilnery Rivera-Figueroa, Glizette O. Arroyo-Morales, Diana T. Medina-Laabes, Roxana Soto-Abreu, Manuel Rivera-Encarnación, Olga L. Díaz-Miranda, Ana P. Ortiz, Katelyn B. Wells, Coralia Vázquez-Otero, Pamela C. Hull

**Affiliations:** 1 Puerto Rico Cancer Control and Population Sciences Division, University of Puerto Rico Comprehensive Cancer Center, PO Box 36302, San Juan, 00936-3027 Puerto Rico; 2 Department of Health Services Administration, Evaluation Research of Health Systems Science Program, School of Public Health, Medical Science Campus, University of Puerto Rico, PO Box 365067, San Juan, 00936-5067 Puerto Rico; 3 Department of Biostatistics and Epidemiology, Graduate School of Public Health, Medical Sciences Campus, University of Puerto Rico, PO Box 70184, San Juan, 00936-8184 Puerto Rico; 4 Association of Immunization Managers, 620 Hungerford Dr. Suite 29, Rockville, MD 20850 USA; 5 Harvard T.H. Chan School of Public Health Dana-Farber Cancer Institute, NCI Postdoctoral Fellow, 450 Brookline Ave., LW 633, Boston, MA 02215 USA; 6 Department of Behavioral Science, College of Medicine, Markey Cancer Center, University of Kentucky, 2365 Harrodsburg Rd, Suite A230, Lexington, KY 40504-3381 USA

**Keywords:** Human papillomavirus, HPV vaccine implementation, School-entry policy requirements, Content analysis, Puerto Rico

## Abstract

**Background:**

In August 2018, Puerto Rico (PR) became the 4th state or territory in the United States to adopt a human papillomavirus (HPV) vaccine school-entry requirement, for students 11–12 years old. Evidence suggests that the content of media coverage may impact people’s perception of HPV vaccine and their willingness to vaccinate. This study aimed to analyze the content of digital news coverage related to the implementation of the policy in PR.

**Methods:**

A content review was conducted of digital media published from January 2017 through December 2018. The content reviewed was carried out in two steps: 1) creating a matrix to summarize each article’s content about the policy and 2) qualitative analysis using a grounded theory approach.

**Results:**

The search resulted in 34 articles obtained from 17 online local and international news outlets that reported the policy's implementation. Analyses showed that 61% of the news articles did not mention the number of required doses, and 79% discussed the new policy concerning cancer prevention. In 2017, news coverage focused mostly on describing the policy, while 2018 coverage focused on controversies surrounding the implementation. Neutral emergent codes included: 1) Description of the policy; 2) Information about HPV related cancers; and 3) General information about HPV vaccine. Negative emergent codes included: 1) infringement to patient and parental autonomy; 2) Hesitancy from the political sector, and 3) Hesitancy from groups and coalitions. Positive content included: 1) knowledge and acceptance of HPV vaccine for cancer prevention; 2) importance of education and protective sexual behaviors; and 3) new vaccination law proposal.

**Conclusions:**

Most of the media coverage in PR was neutral and included limited information related to the vaccine, HPV, and HPV-related cancers. Neutral and negative themes could influence public concerns regarding the new policy, as well as HPV vaccination rates in PR.

## Background

High-risk types of human papillomavirus (HPV) are thought to be responsible for more than 90% of anal and cervical cancers, about 70% of vaginal, vulvar, and cancers of the oropharynx, and more than 60% of penile cancers [1]. Puerto Rico (PR), a United States (US) territory, have documented the burden of HPV infection and HPV-related cancers in this population [2–4]. Studies conducted has reported an increased risk of cervical cancer in this population (compared to mainland US), multiple barriers to cervical cancer screening [5, 6], low awareness of HPV and the HPV vaccine [7], increasing trends of anal cancer [8, 9] and a higher burden in the incidence and mortality from penile cancer in Puerto Rican men compared with other racial/ethnic groups in the US [10]. The significance of these studies led to multiple efforts from the academia, various local community coalitions, the medical community and other health professionals in PR, aiming to increase community engagement and capacity to reduce the burden of HPV-associated cancers in the island [3, 6, 10–17]. All these accomplishments led to an increase in vaccine uptake over time, and thus higher HPV vaccination rates in PR compared to mainland US.

The Healthy People 2030 goal for human papillomavirus (HPV) vaccination for adolescents is an 80% vaccination rate for the complete HPV series (*Up to Date*) in males and females [18]. Estimates from the 2016 National Immunization Survey (NIS), the most recent year available for PR, reported that 75.8% (95% CI: 70.2–80.6%) of children ages 13–15 years in PR had initiated the first dose of HPV vaccine, and 52.8% (95% CI: 46.4–59.0%) were up to date with the HPV vaccine series [19]. When comparing similar estimates (initiation and up to date) with other US states and jurisdictions, PR had the 6th highest HPV vaccination rates in that year [20]. Only 10 out of 64 states and jurisdictions (including PR) had HPV initiation rates higher than 75%.

School-entry requirements are an evidence-based strategy that has been proven effective for increasing coverage rates for other vaccines [21]. In August 2018, PR became the 4th US state or territory to adopt an HPV vaccine school-entry policy, adding the HPV vaccine to the list of required vaccines for school entrance for children aged 11–12 years, known as Law #25 [22]. Three additional states and jurisdictions including Virginia (2008), Washington DC (2009) and Rhode Island (2015) had previously required the HPV vaccine for school entry, and Hawaii subsequently adopted a requirement as of July 2020; although the scope of all the policies enacted so far varies regarding sex, age and number of doses required [23]. Virginia and Washington DC passed the laws through a legislative process, while Rhode Island, PR and Hawaii took a regulatory approach using the authority of the Department of Health.

Studies evaluating the impact of HPV vaccine school entry requirements in Virginia, DC and Rhode Island on vaccination rates have been inconsistent [24, 25], likely due to differences in exemption and enforcement provisions, organizational factors, and parental non-compliance [26, 27]. Countries with successful vaccination programs have acknowledged the importance of monitoring the media as it might shift perceptions and parental motivations to vaccinate, even in the context of a school requirement [28]. The media has been documented as the second most significant source (after health care providers) of information for parents to make vaccination decisions [29]. Documentation of how HPV school entry policies and controversy about them are covered in the news media can help understand the context in which the policies are implemented and in which parents make decisions to comply with the policy or not.

## Methods

The aim of this study was to examine the content of online news coverage related to the HPV vaccine school-entry requirement in PR before and after the first year of the implementation.

### Data collection

**Fig. 1:**
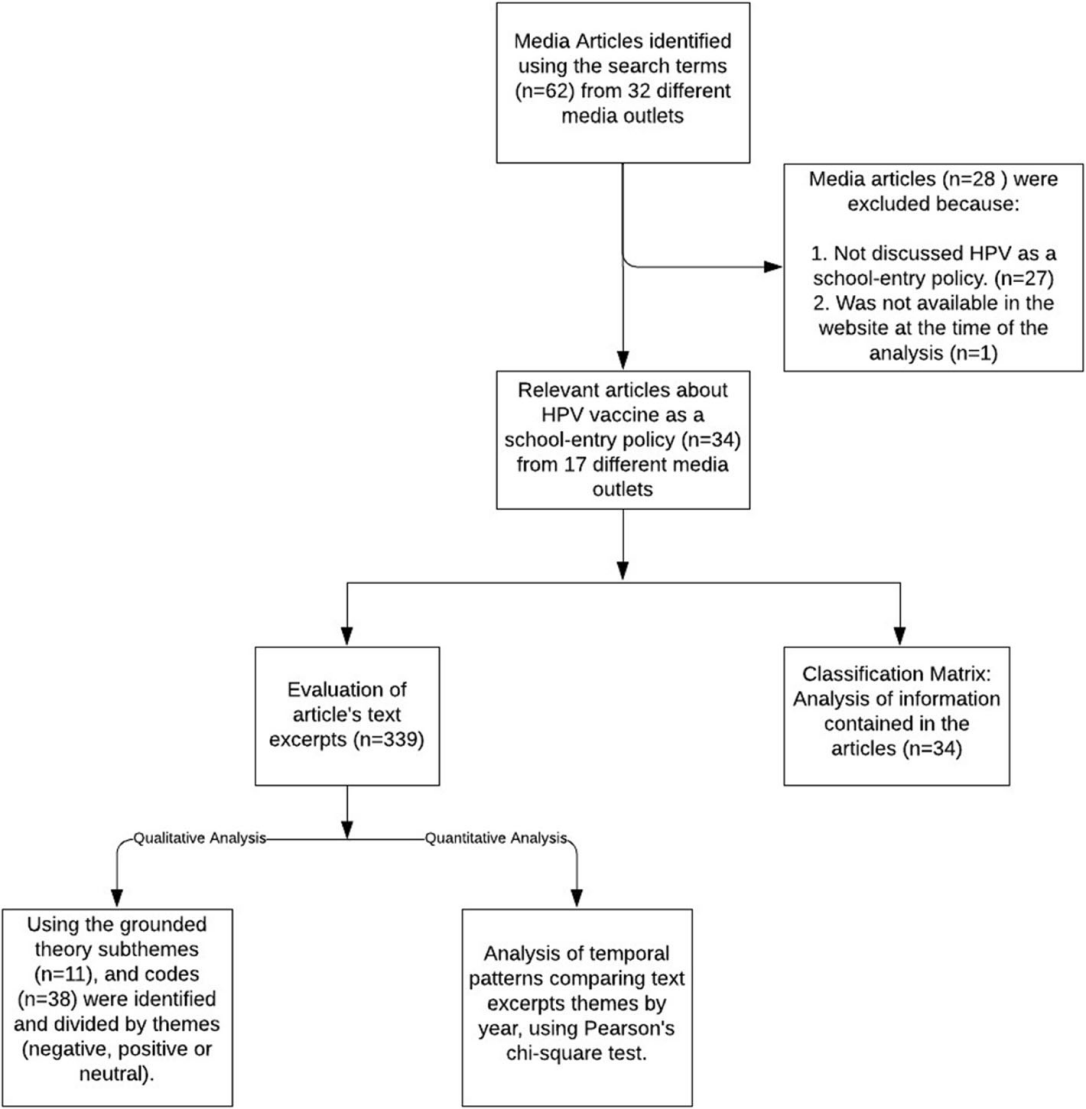
Flow diagram showing inclusion and exclusion strategy

A systematic search was conducted using Google (www.google.com/pr) to locate digital media reports related to the HPV vaccination policy and its implementation in PR from January 2017 to December 2018. Reputable online news sources (such as: endi.com, elvocero.com, metropr.com, noticel.com) who comply with the criteria of reliable sources [30] were included in the analysis. The following search terms (in Spanish) were used to identify the online coverage reports: *virus de papiloma humano* (human papillomavirus)*, VPH* (HPV)*, vacuna OR vacunación contra VPH* (vaccine OR HPV vaccine)*, implementación* (implementation)*,* Puerto Rico (PR). Only articles that address the HPV school entry policy were included in the analysis. Most of the articles reviewed were from outlets in Puerto Rico and written in Spanish; only one was in English. Other exclusion criteria and analysis performed are presented in Fig. 1.

### Data analysis

**Fig. 2:**
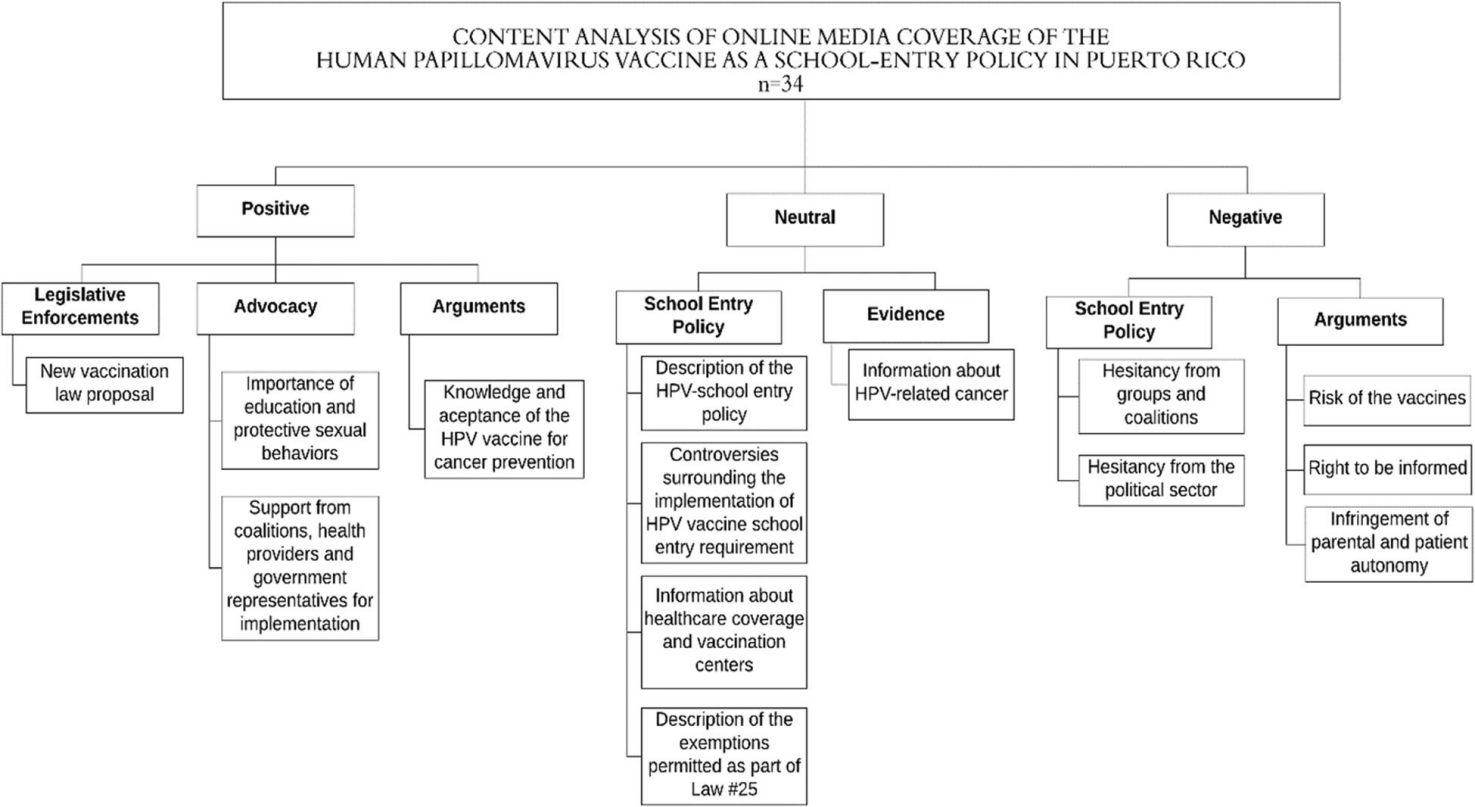
Most emergent codes and subthemes by tones

A series of steps were performed. First, a classification matrix was developed by the research team to identify if the selected news articles contained information regarding: (a) the policy enactment date and (b) groups impacted by the policy (e.g. age group, sex), (c) doses required for the HPV vaccine, the d) HPV vaccine prevention messages (e.g. cancer prevention), and the (e) groups cited (e.g. coalitions, Government agencies). Second, using a modified grounded theory approach [31], members of the research team identified emergent themes in the data. A codebook with definitions of each of the codes identified as well as the main themes was developed. We used the qualitative data analysis software *Atlas.ti* (version 8) to facilitate data manipulation and retrieval during analysis. To identify the key themes, coders independently read the transcripts and identify the data *by tone (level 1), sub themes (level 2) and codes (level 3)* **(**Fig. 2**).** Research staff discussed conceptual differences regarding coding terminology and established a common set of codes [32]. All points of disagreement were resolved through discussion before the remaining articles were analyzed. We compared the frequency of text excerpt by tone and year using the Pearson’s chi-square test. The tests were 2-sided with 0.05 significance levels. The analyses were performed using Stata version 14.1 software (StataCorp LP, College Station, TX). This study was approved by the IRB of the University of Puerto Rico Medical Sciences Campus.

## Results

### Search results

Throughout January 2017 to December 2018, a total of 66 news articles were found referring to the search terms used. Only 35 articles satisfied our inclusion criteria by focusing on the school-entry policy, and one of these was not accessible at the time of the analysis (*N* = 34). Figure 1 summarizes the selection of the news articles and analysis derived from articles studied. A total of 18 (53.0%) news articles were gathered in 2017 and 16 (47.0%) from 2018.The search resulted in 17 online local and international media outlets that reported on news related to the HPV school-entry requirement in PR.

### Descriptive characteristics of article content

Descriptive information about the content of the news articles (*n* = 34) derived from the classification matrix is shown in Table 1. Almost all the news articles reported the date in which the HPV vaccine school-entry requirement took effect (91.2%). More than half of the news articles included information about cervical cancer (58.8%), while other HPV-related cancers were mentioned less often (11.8 to 23.5%). Most articles highlighted the importance of the vaccine for cancer prevention (79.4%), while around a quarter of the articles discussed HPV as a sexually transmitted infection (STI) (26.4%). Most of the articles did not discuss the number of doses needed to complete the immunization series (38.2% included this information); likewise, information regarding the accessibility of the vaccine was not provided frequently (23.5%). Government agency representatives, followed by coalitions and groups against the implementation, were mostly cited as spokesperson.

**Table 1:** Content of the Online Media Articles Analyzed Regarding HPV Vaccine School-Entry Requirement: 2017–2018 (*n* = 34 Articles)

Topics discussed	N	Percent (%)
*HPV-associated cancers*		
Cervical cancer	20	58.8
Oropharyngeal cancer	8	23.5
Penile cancer	7	20.6
Vulvar/vaginal cancer	7	20.6
Anal cancer	4	11.8
Effective date of mandate	31	91.2
Number of doses that are recommended for complete HPV vaccination	13	38.2
Concerns about the efficacy and safety of the vaccine	19	55.9
Highlighted the importance of the vaccine for cancer prevention	27	79.4
Referred to HPV as a STI	9	26.5
Information regarding HPV vaccine accessibility and health coverage	8	23.5
Groups Cited		
Coalitions Groups Against the Implementation	11	32.4
Government Agency Representatives	29	85.3
Health Professionals	10	29.4
Data and Statistics Institutes	14	41.2
Coalitions Groups In favor of the Implementation	4	11.8
Others	3	8.8

### Neutral media

Most of the media reports published were neutral (58.6%), being the most emergent: (1) description of the HPV-school entry policy; (2) information about HPV-related cancers; (3) general information about the HPV vaccine; (4) information about health care coverage and vaccination centers; (5) controversies surrounding the implementation of HPV vaccine school-entry requirement; and (6) description of the exemptions permitted as part of Law #25. The PR Secretary of Health was the most common spokesperson who disseminated this information. Other news articles provided data from governmental or cancer registry sources, highlighting the burden of cervical cancer and the disparities in cervical cancer incidence in PR in comparison to mainland US. They also stated that these data were fundamental reasons for the decision to implement the HPV vaccine school-entry requirement. Fig. 2 shows the subthemes and the most emerging codes.

#### Description of the policy (11.8%)

Most of the neutral text excerpts disseminated in the media described the policy’s announcement when it was going to take place and the required ages.

*“All students between the ages of 11 and 12 must be vaccinated against the Human Papilloma Virus (HPV) by next year, the government of Puerto Rico ordered on Monday.”*

-Journalist

#### Information about HPV related cancers (9.4%)

Other text excerpts provided data from governmental or cancer registry sources, highlighting the burden of cervical cancer and the disparities in cervical cancer incidence in PR versus mainland US, and stated that these data were fundamental reasons for the decision to implement the HPV vaccine school-entry requirement.

#### Controversies surrounding the implementation of HPV vaccine school-entry requirement (5.6%)

Results from the content analysis highlighted controversies a year prior to the implementation, with disagreement among groups in favor of and against the HPV vaccine school-entry requirement. This text excerpts discuss the arguments that questioned the implementation of the HPV vaccine as a requirement and the lack of compliance with the policy.

*“The vaccine against human papillomavirus (HPV) stirred controversy again since its release to the market 12 years ago, a period in which groups in favor and against [the vaccine] have deliberated on its benefits and risks.”*

- Journalist

*“On the one hand, some people applaud the Department of Health initiative so that boys and girls of 11 and 12 years old are inoculated against this disease, after being included in the list of vaccines required for school entry as of August 2018. However, others question the government’s obligation to promote a vaccine that they classify as unsafe and effective due to the experience observed in other countries. Adverse effects have been reported and created organizations of parents against it.”*

- Journalist

#### Description of the exceptions process (4.7%)

These text excerpts discussed details regarding the exemption process, explaining that PR only accepts medical and religious exemptions provided via a vaccine exemption affidavit that has to be submitted to the child’s school [22].

*“The Secretary of Health … said the measure applies to both public and private schools. Parents may disregard the measure for medical or religious reasons if they present an affidavit or a medical certificate.”*

-Journalist

Other neutral codes discussed information regarding coverage of the cost of vaccination through the Vaccine for Children (VFC) Program and accessibility of the vaccine via the government health insurance program (5.9%). Lastly, neutral themes referred to the recommendation of HPV vaccine together with other vaccines (i.e., recommending the HPV vaccine in the same way and at the same visit as Tdap and meningococcal vaccines) (1.5%).

### Negative media

The most common emerging negative codes are the following: 1) infringement of parental and patient autonomy; 2) hesitancy from the political sector (e.g. legislators); 3) hesitancy from groups and coalitions; 4) risk of the vaccine (safety and efficacy); and 5) right to be informed.

#### Infringement of parental and patient autonomy

A total of 17 (5.0%) of the excerpts studied discussed this code, being one of the most emergent topics within negative media*.* Overall, articles reported negative view*s* towards the mandate of the vaccine (not HPV vaccination itself).

*… “vaccines against HPV are a tool for cervical cancer prevention; however, the vaccine should not be mandatory. These stories were mostly presented by coalitions and organizations spokespersons in which they described the need to respect parental autonomy with regards to medical decision-making for their children”.*

-Spokesperson from *local coalitions statement*

#### Hesitancy from groups and coalitions (5.0%)

The negative tone codes highlighted the hesitancy of various groups against the new HPV vaccine school entry policy, which stated different reasons for their hesitancy (Table 2). Some of the groups were not against vaccines, but they expressed hesitation against the mandate.

**Table 2:** Groups against the HPV vaccine school-entry requirement and their reasons

Group	Reasons against HPV vaccine school-entry requirement
Family coalition	The vaccine only protects against the most common virus strains and questions its effectiveness.
Feminist groups	Did not object to the vaccine but question the obligatory nature of the policy since the vaccine has been controversial. Recommend that it be accompanied by vaccine education and sex education. State that each family must decide freely in their health strategies.
Local coalitions against HPV school entry requirement	Violates the rights of parents in deciding on the care of their children, and they have the right to be informed of the risks of any medical treatment. They understand that the government is obstructing education with this requirement given the high number of vaccines that are imposed annually as part of the school-entry policy and support of the 1576 bill

#### Risk of the vaccine (safety and efficacy) (2.9%)

Worry about vaccine safety and discussion of the secondary effects associated with the HPV vaccine were also discussed in the online media outlets. This concern was mostly expressed by coalition groups against the implementation of this vaccination policy.

*“This project [1303] does not even mention the risks inherent to vaccination. [When you] read [the project] it seems that the vaccines are made from holy water,”*

-Quote from local coalition spokesperson against HPV school-entry requirement

*“We are greatly concerned about the lack of orientation on the risks of vaccination. This practice leads to an uninformed or dishonorable consent [without the proper orientation] can put risk health and life at risk”, said the organizers.*

-Quote from local coalition spokesperson against HPV school-entry requirement

Information in the news articles noted that the HPV vaccines are a cervical cancer prevention tool; however, they highlighted opinions of some groups that the vaccine should not be mandatory (3.8%). In summary, those coalitions, and groups state that each family, after being properly informed about the vaccine’s advantages and risks, should freely decide which of these strategies they want to assume for them and their family health.

*“[This requirement] needs to be assessed carefully. If it is proven [that the vaccine] is effective and safe, I don't see any problems as long as [this requirement] will not surpass parental choice”.*

-Spokesperson of a family organization against HPV school entry requirement

#### Right to be informed (2.7%)

Like the prior code, most of the interviewee were not against the HPV vaccine but acknowledged the importance of advocating for understanding the risk and benefits of the vaccine and discussed the right of the parents to be informed.

*“We are not against vaccines, but what is being [administered] to our children has benefits and risks. It is our right as parents to be informed”.*

-Local coalition spokesperson against HPV school entry requirement

### Positive media

Four codes emerged that were positive in tone in the online articles: 1) knowledge and acceptance of the HPV vaccine for cancer prevention; 2) importance of education and protective sexual behaviors; 3) new vaccination law proposal; and 4) support from coalitions, health providers and government representatives for implementation.

#### Knowledge and acceptance of the HPV vaccine for cancer prevention (3.8%)

Many positive tone text excerpts presented prior to and after the implementation of the HPV vaccine school-entry requirement in PR addressed the importance of HPV vaccination for cancer prevention.

*“I understand that this is something positive because it is a vaccine that has been used since 2006 in Puerto Rico and the United States … a vaccine that is going to prevent cancer, a very common cancer, that when you see the data it affects many of our patients. It has a very great burden on our patients, so I understand that it is a positive thing.”*

-Pediatrician

*The importance of educate* the population of the benefits of this vaccine were documented.

*“The message that must be given to the people of Puerto Rico, is that [HPV related cancers] are totally preventable. When you get vaccinated, it prevents the development of cancer, and as I said before, we have to protect our children, our adolescents”*

- Department of Health Representative

*“Yes, a lot of people don't make the link between the virus and cancer. What they have to understand is that it is a vaccine that prevents cancer, it is extraordinary … ”*

–News Reporter

#### New vaccination law proposal (1.8%)

Misleading information about the HPV vaccine mandate was disseminated in the media about the bill 1303 (a legislative proposal in PR which was intended to create a new law for children and students aiming to strengthen disease prevention through vaccination among these groups [33]). The quotes were unclear if they were in effect talking about the new bill proposed 1303 or the HPV school-entry requirement that would be effective in August 2017.

#### Support from coalitions, health providers and government representatives for implementation (1.5%)

Statements that stressed the importance of HPV vaccination were mostly driven by personal support of the vaccine, whereas coalitions, and other medical and community-based organizations emphasized the importance of this vaccine school-entry requirement for cancer prevention, addressing the burden of HPV and HPV-related cancers in their messages.

*“Experts urge parents to vaccinate their children by the beginning of the 2017-2018 school year and, if so, complete the corresponding vaccination processes, according to a press release for PR Vaccination Coalition and HPV Advisory Panel.”*

-Journalist

### Temporal patterns

**Fig. 3:**
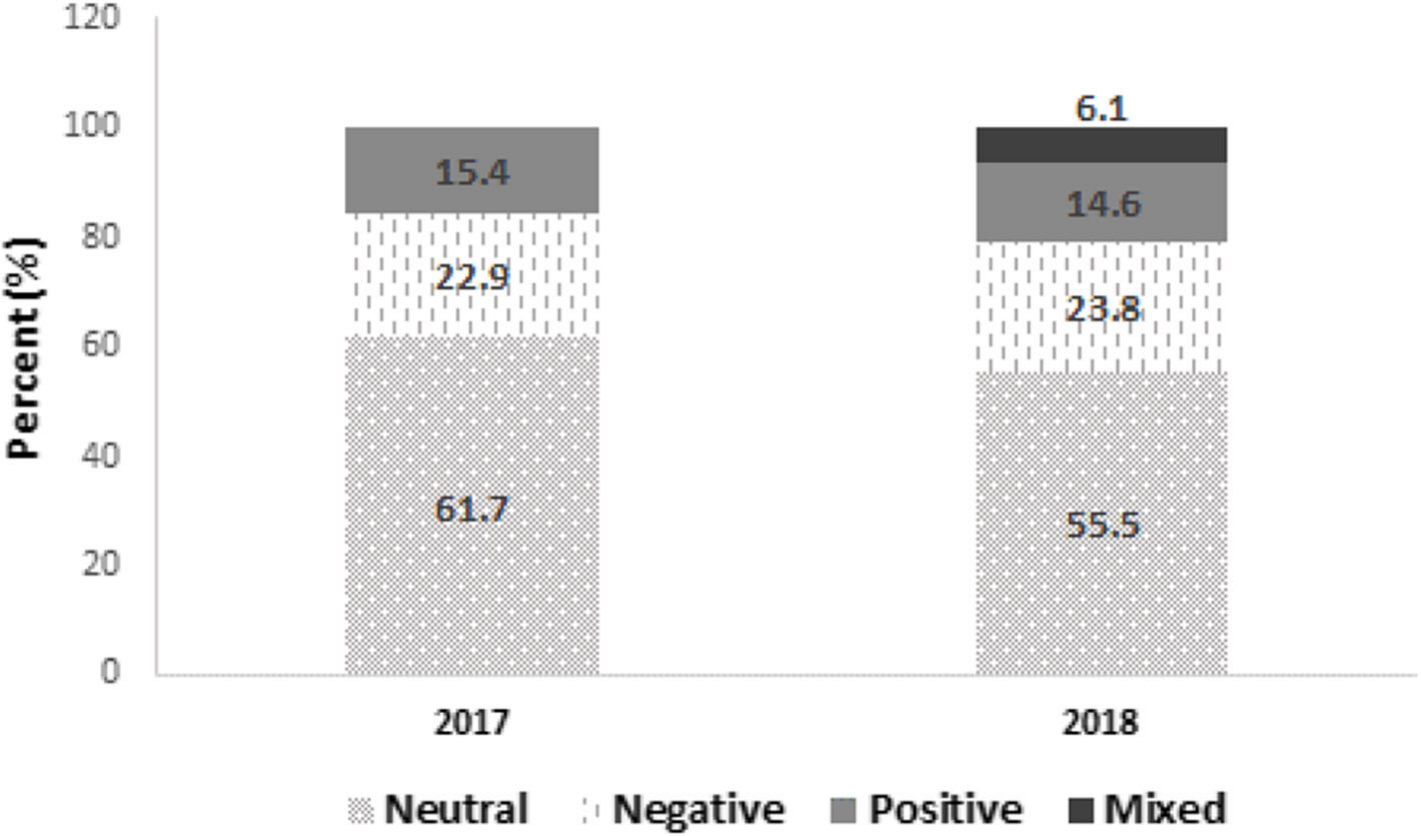
Distribution of theme (*n* = 339) from news articles related to HPV vaccine school-entry requirement in PR

Across the 17 articles studied, a total of 339 text excerpts were coded as having a positive, negative, and neutral theme. There were some excerpts that had more than one theme (mixed theme). Figure 3 shows a distribution of the themes by year. For both years (2017 and 2018), the highest proportion of text excerpts were neutral (61.7 and 55.5%, respectively) towards the HPV vaccine school-entry requirement, followed by negative themes. In 2018, we observed an increase of 0.9% on negative themes compared with 2017. No significant differences across the years were observed by themes (*p* > 0.05).

## Discussion

Previous research has documented how mass media might influence public risk perceptions and decisions about immunization and HPV, but few have examined the context before and prior the implementation of an HPV school entry requirement [28, 29, 34–37]*.* The results from our study showed that although information regarding the HPV vaccine school-entry requirement was disseminated prior and during the implementation, most of the information during this period was neutral. Changes in themes during this period as well as controversies during the two-year period of the enactment might have led to confusion of the public about the implementation of this mandate.

Content analysis of the media’s representation of the HPV vaccine worldwide demonstrates that the themes associated with the vaccine are variable, ranging from negative to neutral to positive [38–42]. A notable aspect from the media discourse in PR before and after the HPV vaccine school-entry requirement was that media coverage appeared to be more neutral in content than in most countries. Although it is important to acknowledge that the methodology used in different studies varies, a study conducted in Romania showed that (28.0%) of the media reports were negative, in the US 14.4% of the media content reported had a similar tone, and a study in Japan which studied newsprint media reported 23.3% [42–44]. Most of the negative context of the themes analyzed in our study were driven towards the risk of the vaccine, infringement to patient autonomy, right to be informed, and lack of education about HPV and the vaccine. Similar findings were noted in a another study, were concerns regarding vaccine safety and parent’s right to choose, were commonly cited as arguments against the HPV vaccine school-entry requirement [45]. Another study explored news coverage in states and jurisdictions in which the mandate was implemented, highlighted similar topics [46]; and discussed how this discourse might negatively impact the HPV vaccine school-entry requirement but the adolescent immunization schedule as well.

It was noted in our study that the DOH provided most of this neutral content. Scientists or academics were not included as part of this discussion, which might have been a missed opportunity that could have brought robust positive statements in favor of the vaccine and the mandate [47]. Movement in opposition mostly stress HPV vaccine safety, patient and parent autonomy rights; those are legitimately issues that can be promoted in educational campaigns and the media. When compared to anti-vax movements worldwide, although similar in scope, the magnitude and impact of these groups on Puerto Rico might differ, which could translate in higher HPV vaccine acceptance rates prior to the requirement and in the increase in other vaccine-preventable diseases as well [48–50].

Our analysis showed a shift in the content of the online media from informational (neutral) prior to the implementation to a negative content of information from a coalition against the implementation. This shift was probably driven by the one-year lag period, which was led to promote the new HPV vaccine school-entry requirement. During this period, legislators requested hearings to be informed regarding the safety and efficacy of the vaccine. Since PR’s immunization law authorizes the Secretary of Health to require vaccinations for school-entry, the participation of the Legislature seems to have brought confusion and provoked controversies regarding the implementation of the HPV vaccine school-entry requirement. This also led to the development of a bill, which included philosophical exceptions [51]. Although this later bill (1576) was not approved, this period and the mixed information disseminated by the media at the time of the HPV vaccine school-entry requirement might have triggered confusion about the actual implementation.

This study adds to the literature on the main themes disseminated prior to the implementation of HPV vaccine school-entry requirements, using PR as an example, and to research on the media coverage of mandates, which may in fact, create a backlash to government-sponsored public health programs [46]. However, this study had some limitations. We used a selection of only online news articles focused on HPV vaccine mandates rather than including articles where the HPV vaccine was mentioned, limiting the analysis without news focusing only in HPV and HPV vaccine, which could affect the amount of news with positive and/or neutral content. Also, the small sample of online news articles reviewed might limit the depth of the qualitative analysis of the data, and robustness of the interpretation. This understandably make this initial effort worth of a prospective evaluation of content analysis several points after the implementation of the school-entry requirement. The timeframe established for inclusion criteria to gather the news, which was less than 24 months, and the fact that the media coverage was focused on the school entry policy for HPV vaccination, could also have affected the sample size. Most of the previous studies had an extended timeframe (more than two years) and had a broader HPV-related coverage search [46]. Existing search databases were considered for content analysis. However, since access to these databases was restricted and required additional cost and registration (e.g., LexisNexis), and might not contain a broad array of news content in Spanish; we continued with the analysis of digital newspapers with free access. In addition to this, in September 2017, Hurricane Maria harmed the dissemination and publication of information in the news media due to the state of emergency in PR. Nonetheless, the HPV vaccine requirement and legislative activity after the implementation started as planned.

Despite these limitations, this effort of understanding the context of the media regarding news related to the HPV vaccine school-entry requirement implementation in important for future public health efforts, particularly given recent changes to the requirement entering the 2019 academic period (expanding to 11–14 years old during the 2019–2020 academic period). However, this decision was also later impacted by environmental disasters in January 2020 (earthquake and continuous seismic activity primarily in the South-west of the island) and the COVID-19 pandemic that might have impacted immunization uptake, hesitation towards the HPV vaccine and might change the narrative and online media discourse on immunization.

## Conclusion

This study explored the content of online media prior and during the first year of implementation of PR’s HPV vaccine school-entry requirement. Vaccine safety and autonomy concerns were common. Detailed information about the vaccine school-entry requirement as well as education on the importance of this policy for the prevention of HPV-related cancers was frequently missing, which could lead to an incomplete picture or lack of understanding of the public health importance of this mandate. Due to the impact that media can have on important immunization strategies, continued monitoring and active participation of coalitions and scientists in favor of these mandates are important. Scientists and providers should insert in the discussion developing content to disseminate the benefits of the vaccine, specifically to neutralize the negative content. Other media sources, such as radio, television, blogs, podcasts, among others, can contribute another aspect or a different level of acceptance to analyze how this content in the analysis can influence the perspective of users related to the HPV vaccine. Future research should continue to monitor news media depictions of the HPV vaccine to assess which themes or controversies remain a pronounced theme of coverage or whether the media ultimately presents the HPV vaccine as an important routine public health service.

## Data Availability

The datasets used and/or analyzed during the current study are available from the corresponding author on reasonable request.

## Abbreviations

HPV: Human papilomavirus
VPH: Virus del papiloma humano
PR: Puerto Rico
US: United States
NIS: National Immunization Survey
STI: Sexually transmitted infections
